# Case Report: Paraneoplastic Tumefactive Demyelination Associated With Seminoma

**DOI:** 10.3389/fneur.2022.946180

**Published:** 2022-07-11

**Authors:** Wataru Shiraishi, Takeru Umemura, Yuuki Nakayama, Yui Yamada, Masahiro Shijo, Tetsuya Hashimoto

**Affiliations:** ^1^Department of Neurology, Kokura Memorial Hospital, Kitakyushu, Japan; ^2^Department of Internal Medicine, Shiraishi Internal Medicine Clinic, Nogata, Japan; ^3^Department of Neurosurgery, Kokura Memorial Hospital, Kitakyushu, Japan; ^4^Department of Urology, Kokura Memorial Hospital, Kitakyushu, Japan; ^5^Department of Pathology, Kokura Memorial Hospital, Kitakyushu, Japan; ^6^Department of Internal Medicine, Fukuoka Dental College Medical and Dental Hospital, Fukuoka, Japan; ^7^Department of Neuropathology, Graduate School of Medical Sciences, Kyushu University, Fukuoka, Japan

**Keywords:** anti-amphiphysin antibody, COVID-19 vaccination, demyelinating disease, paraneoplastic syndrome, seminoma, stereotactic biopsy, steroid therapy, tumefactive demyelination

## Abstract

Paraneoplastic tumefactive demyelination (TD) is a rare disorder of the central nervous system that can be challenging to diagnose. Here, we describe a 32-year-old Japanese man with a TD associated with testicular seminoma. He presented with symptoms of right-sided motor and sensory impairment 2 days after vaccination for coronavirus disease 2019 (COVID-19). Brain magnetic resonance imaging (MRI) showed a high-intensity lesion in the left internal capsule. He had a 3-year history of enlargement of the left testicle. Blood examination showed tumor marker elevation and the presence of anti-amphiphysin antibodies. Whole-body computed tomography (CT) revealed mass lesions in the left testicle and enlargement of the retroperitoneal lymph nodes. Radical orchiectomy was performed. As the pathology showed testicular seminoma, chemotherapy was administered. After surgery, his neurological symptoms deteriorated. MRI revealed that the brain lesion had enlarged and progressed to a tumefactive lesion without gadolinium enhancement. The cerebrospinal fluid (CSF) examination was normal without pleocytosis or protein elevation. Steroid pulse therapy was added; however, his symptoms did not improve. A brain stereotactic biopsy was performed and the sample showed demyelinating lesions without malignant cells. As the initial corticosteroid therapy was ineffective, gamma globulin therapy was administered in parallel with chemotherapy, and the clinical symptoms and imaging findings were partially ameliorated. TD seldom appears as a paraneoplastic neurological syndrome. In addition, there are few reports of COVID-19 vaccination-associated demyelinating disease. Clinicians should recognize paraneoplastic TD, and the further accumulation of similar cases is needed.

## Introduction

Tumefactive demyelination (TD) is uncommon and its diagnosis is often difficult. Most instances of TD occur in the context of multiple sclerosis (MS), mainly associated with fingolimod and natalizumab (1). TD resembles a malignant tumor, and sometimes a biopsy is required for an accurate diagnosis. If TD appears as an isolated lesion without the context of MS, TD does not usually progress to MS, and clinicians should consider other causes, such as malignancy-associated TD (2). Here, we describe a pathologically confirmed case of TD as a phenotype of seminoma-associated paraneoplastic disorder and post-COVID-19 vaccination. In our case, the clinical symptoms and imaging findings were partially improved by treatment of the seminoma and immunotherapy. This is the sixth case of pathologically confirmed TD associated with seminoma.

### Case Presentation

A previously healthy 32-year-old Japanese man was admitted to our hospital due to right hemiparesis. He was aware that his left testicle had started to enlarge 3 years earlier, but he had not sought medical attention. He received a second vaccination for severe acute respiratory syndrome coronavirus 2 (SARS-CoV-2) (Spikevax® Moderna) in his left deltoid muscle. Two days after vaccination, he developed right hemiparesis and was admitted to our hospital.

**Figure 1:**
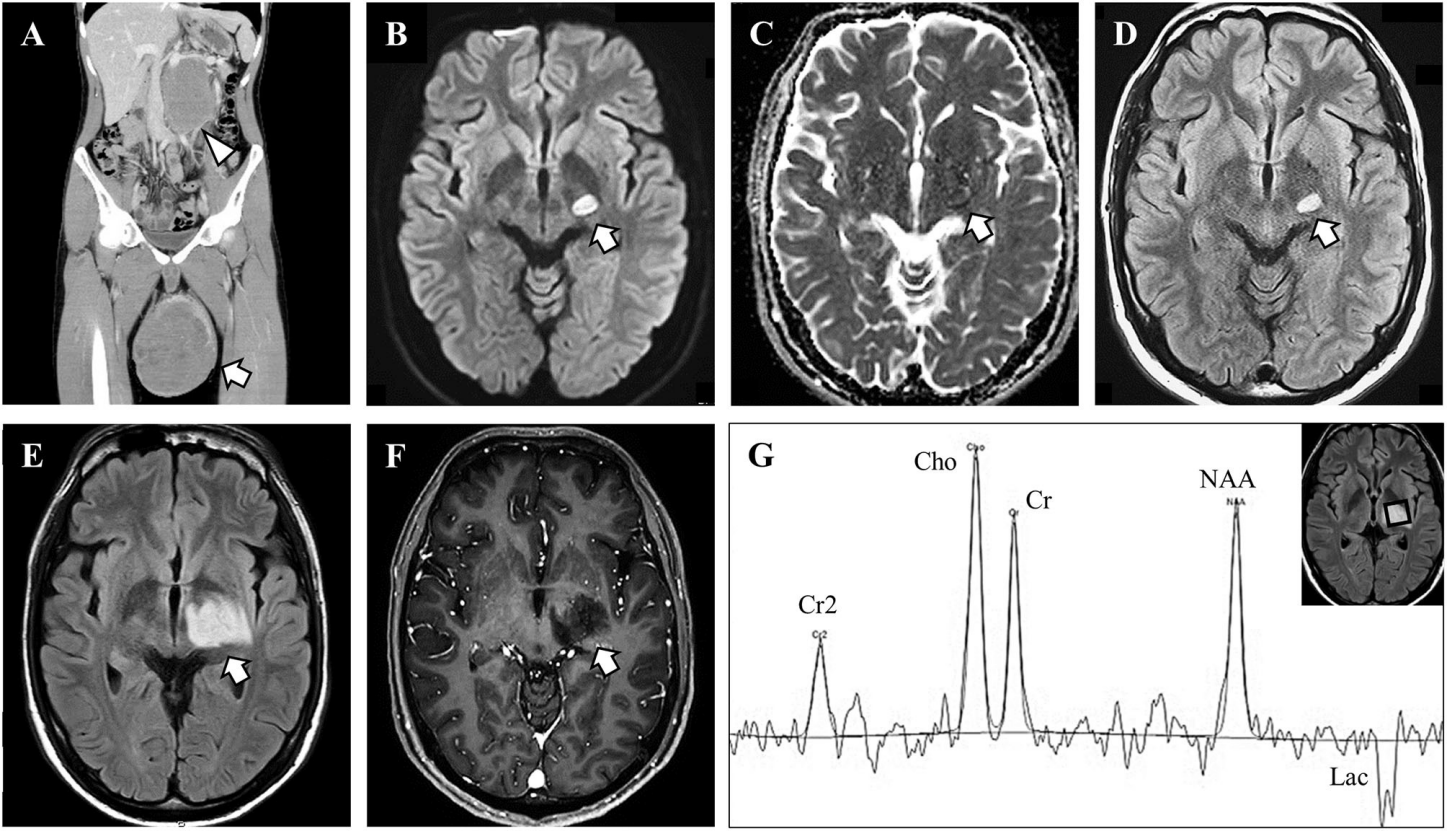
Imaging and magnetic resonance spectroscopy findings. **(A)** Body trunk computed tomography showed enlargement of the left testicle with heterogeneous content (arrow) and retroperitoneal lymph node (arrowhead). **(B–D)** Brain magnetic resonance imaging (MRI) on admission showed a high-intensity lesion in diffusion-weighted and fluid-attenuated inversion recovery (FLAIR) images, with iso-intensity in apparent diffusion coherence images. **(E, F)** The brain lesion enlarged afterward, but with no gadolinium enhancement. **(G)** Magnetic resonance spectroscopy showed high choline (Cho) peak, preserved N-acetyl aspartate (NAA) peak, and lactate (Lac) peak. Cr, creatine.

On physical examination, he was alert and his body temperature was normal (36.4°C). He showed no headache or neck stiffness. He had no aphagia, apraxia, or agnosia. He showed right hemiparesis with a manual muscle test (MMT) score of 4/5, right hemihypesthesia, and right-sided hyperreflexia. His left testicle was enlarged to 15 cm in diameter. Blood examination showed elevated lactate dehydrogenase (3,520 U/L; normal: 124–222 U/L) and human chorionic gonadotropin (58.1 IU/L; normal: <2.7 IU/L) levels, but alpha-fetoprotein was within the normal limit (3.1 ng/mL; normal: <10 ng/mL). He was negative for anti-nuclear, -neutrophil cytoplasmic, and -SS-A antibodies. Among anti-paraneoplastic antibodies, he was positive for anti-amphiphysin antibodies by an immunoblotting method. He was negative for anti-CV2, -PNMA2, -Ri, -Yo, -Hu, -recoverin, -SOX-1, -titin, -zic4, -GAD65, and -Tr antibodies. We have not measured kelch-like protein 11 antibodies (3). Anti-aquaporin 4 antibody and anti-myelin oligodendrocyte glycoprotein antibody were negative. Whole-body CT showed enlargement of the left testicle and retroperitoneal lymph nodes (Figure 1A). Brain MRI revealed a T2 high-intensity lesion in the left internal capsule (Figures 1B–D) without gadolinium enhancement or a mass effect, which did not suggest brain metastasis.

At first, the brain lesion was suspected to be an infarct associated with a malignant tumor. The testicular mass was suspected to be a malignancy based on the retroperitoneal lesions and the serological tumor marker findings. Radical orchiectomy was performed, and after surgery, aspirin was administered as prophylaxis for brain infarction. The testicular tumor was pathologically diagnosed as a seminoma. At 3 days after orchiectomy, the right-side paralysis worsened to an MMT score of 1/5, and right hemianopsia appeared. Follow-up MRI revealed enlargement of the brain lesion with invasion into the left optic tract. The brain lesion presented with slight edema and a mass effect showing a high-intensity lesion on fluid-attenuated inversion recovery and diffusion-weighted imaging. Gadolinium enhancement was still absent (Figures 1E,F). MR spectroscopy revealed an increased choline peak and an abnormal lactate peak, but the N-acetyl aspartate peak was preserved (Figure 1G). On suspicion of brain metastasis, encephalitis, or demyelinating lesions, a lumbar puncture was performed. CSF analysis demonstrated normal protein level (.37 g/L; normal:.15–.45 g/L) and cell count (1 cell/μL; normal: <10 cells/μL). The IgG index was also normal (.42; normal: < .72) and oligoclonal IgG bands were absent. CSF cytopathology showed no malignant cells.

**Figure 2:**
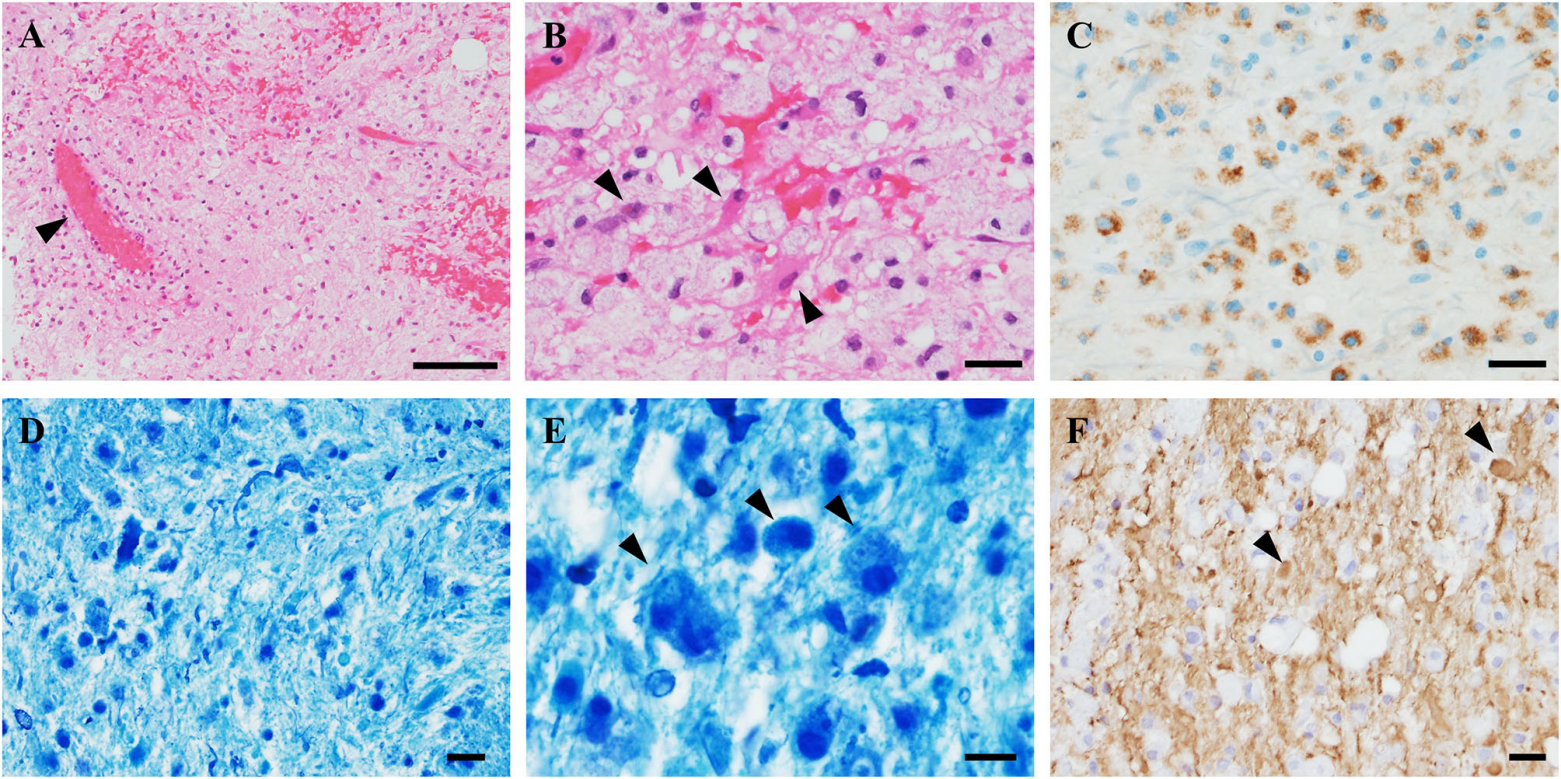
Pathological findings of the brain biopsy. **(A)** Hematoxylin and eosin (HE) stains showed gliosis with widespread infiltration of inflammatory cells without neoplastic tissue. Perivascular inflammation was absent (arrowhead). **(B)** High-magnification HE showed diffuse foamy macrophage infiltration associated with reactive astrocytes (arrowheads). **(C)** CD68-immunostaining revealed clusters of macrophages. **(D,E)** Klüver–Barrera staining demonstrated myelin loss with myelin-laden macrophages (arrowheads). **(F)** Phosphorylated neurofilament immunostaining revealed axonal fragmentation and spheroids (arrowheads). The axonal loss was milder than myelin loss. Scale bars: **(A)** 100 μm. **(B–E)** 20 μm. **(F)** 10 μm.

As treatment for the seminoma, we started chemotherapy (bleomycin, etoposide, and cisplatin). We added corticosteroid therapy (1,000 mg/day methylprednisolone for 3 days) for the brain lesion, but there was no neurological or MRI improvement. We performed a stereotactic brain biopsy from the left thalamus because the brain lesion showed tumor-like enlargement even after corticosteroid therapy. The sampled specimen consisted of gliotic brain tissue with marked myelin loss and infiltration of inflammatory cells, including numerous macrophages, however, neither malignant tissue nor infarct lesion, was observed (Figure 2). The CD68-immunopositive macrophages were widely distributed without perivenous clustering. Some CD68-positive cells may be microglia, but it is difficult to differentiate by immunostaining. The nodular formation of CD 68 positive cells was absent, suggesting there were no microglial nodules. CD3 staining showed scarce infiltration of lymphocytes, so we did not perform CD8 immunostaining. Although axonal loss and degeneration were moderately observed, these findings of axonopathy seemed relatively mild than the degree of myelin loss. Together with these findings, we diagnosed the lesion as a TD, probably due to testicular seminoma.

**Figure 3:**
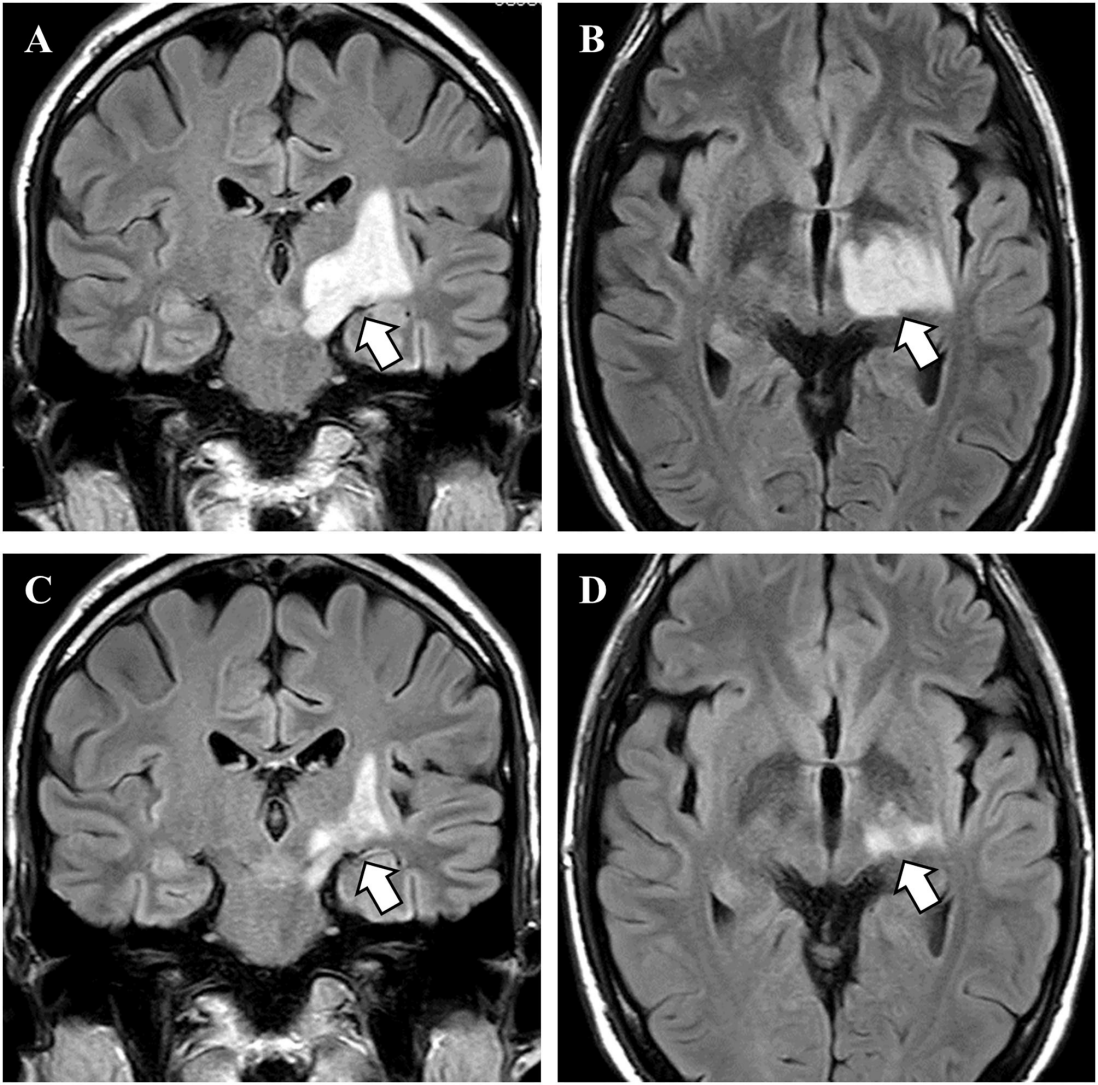
MRI findings before and after treatment. **(A,B)** Before treatment, brain magnetic resonance imaging showed a high-intensity lesion in the left basal ganglia (arrows). **(C,D)** After chemotherapy and immunotherapy, the brain lesion was reduced in size (arrows).

As disease onset occurred 2 days after COVID-19 vaccination, the post-vaccination demyelinating syndrome was also suspected. In addition to chemotherapy, we added second and third corticosteroid pulse and intravenous immunoglobulin therapy. After four courses of bleomycin, etoposide, and platinum chemotherapy, enlargement of the retroperitoneal lymph nodes was reduced, and serum seminoma markers (lactate dehydrogenase and human chorionic gonadotropin) were decreased to within the normal range. After immunoglobulin therapy, the neurological symptoms showed partial improvement, and the right hemiparesis recovered to an MMT score of 3/5. Plasma exchange was not performed because it was anticipated to attenuate the effect of chemotherapy. The hemiparesis and hemianopia remained, and he was referred to a rehabilitation hospital with MRI amelioration (Figures 3A–D).

## Discussion

Here, we report a case of a tumefactive demyelinating brain lesion accompanied by testicular seminoma. There are six previous case reports of TD associated with seminoma (4–9), of which five were diagnosed pathologically. To the best of our knowledge, this is the sixth pathologically diagnosed case report of paraneoplastic TD associated with seminoma. Not like previous cases, we could lead to the correct diagnosis before the lesion expands to the subcortical white matter because we confirmed the pathology by thalamic stereotactic biopsy.

Tumefactive demyelination (TD) mainly occurs in connection with MS and is commonly associated with disease-modifying drugs, such as fingolimod and natalizumab (1). When a demyelinating lesion is isolated and not associated with MS, neurologists should investigate other causes, including malignancy-associated TD (2). We could not rule out metastasis or malignancy because the brain lesion increased in size despite the initial corticosteroid therapy and radical orchiectomy. A brain biopsy eventually led us to the correct diagnosis of TD, and we administered further immunotherapy, which resulted in a partial response. The MRI findings of TD are characteristic. Algahtani et al. reported that minor edema, a mass effect, open-ring enhancement, dilated veins, restricted diffusion, and low perfusion on brain MRI, and an increased choline peak, a relatively high N-acetyl aspartate peak, and an abnormal lactate peak on MR spectroscopy are highly suggestive for TD (1). Diffusion-weighted imaging is reported to show peripheral diffusion restriction with increased apparent diffusion coefficient, and perfusion imaging shows low relative cerebral blood volume (7). These imaging findings and careful management may lead clinicians to the diagnosis of TD without biopsy. Our case presented with a small mass effect without gadolinium enhancement on MRI. In addition, MR spectroscopy showed an increased choline peak, a preserved N-acetyl aspartate peak, and a lactate peak, which are consistent with TD. Sometimes, TD shows disease progression despite steroid therapy (8). Therefore, it is essential to note that resistance to initial steroid therapy does not always rule out demyelinating disease.

There are six previous case reports of TD with seminoma (Table 1). In all cases, the seminoma was discovered simultaneously or after the neurological symptoms had developed. All of these cases, except for the case of Plotkin et al., had pathologically confirmed demyelinating lesions. The brain lesions were predominantly in the white matter and corpus callosum with a tendency to increase in size. Concerning autoantibodies and paraneoplastic neuronal antibodies, only one case (6) was positive for autoantibodies, but all of the other cases were negative or not screened. Among these reports, Thebault et al. attempted serum antibody screening by immunofluorescence immunohistochemistry of rat brain sections and live neuronal cultures, but did not identify specific binding (9). Our case was positive for serum anti-amphiphysin antibodies. The most common neurological manifestations caused by anti-amphiphysin antibodies are stiff person syndrome, ataxia, neuropathy, and neuronopathy (10). In addition, an association between spinal lesions and the presence of anti-amphiphysin antibodies has been reported (11). However, to the best of our knowledge, intracranial demyelinating diseases associated with anti-amphiphysin antibodies, such as the present case, have not been reported. Therefore, it is difficult to conclude that there is an association between the presence of anti-amphiphysin antibodies and the pathomechanisms of this case. Also, there is the possibility of false-negative because generally, amphiphysin antibodies are not associated with seminoma (10, 12). Recently, new antibodies were discovered in cases of encephalitis complicated by seminoma (3). This kelch-like protein 11 antibodies positive encephalitis shows progressive brain-stem, cerebellar, or rhombencephalitis. We did not measure this antibody because the phenotype is different from our case. Further studies may detect antibodies shared by demyelinating diseases associated with seminomas.

**Table 1:** Previous cases of paraneoplastic tumefactive demyelination accompanied by seminoma.

**Case**	**Age (years)**	**Sex**	**Symptoms**	**Imaging findings**	**Biopsy**	**Antibodies**	**Treatment**	**Response**	**Author**
1	41	M	Depression, difficulty with concentration and memory.	Decreased T1-low and T2-high signals in the occipital lobes and corpus callosum, with some peripheral contrast enhancement.	Macrophage infiltration, reactive astrocytosis, demyelination, and preserved axons.	Negative for anti-Hu, –Yo, and –Ri antibodies.	Oral corticosteroid therapy and radiation to the seminoma.	Good response.	Jaster JH
2	54	M	Confusion and memory loss.	T1-low and T2-high signals on the corpus callosum and parieto-occipital white matter. Minimal mass effect and no contrast enhancement.	Foamy macrophages and reactive astrocytes. Complete myelin loss and moderate axonal loss.	Not assessed.	Chemotherapy and dexamethasone.	Partial response. Memory deficit remained.	Wong K
3	37	M	Left facial numbness and left-sided ataxia.	T1-low and FLAIR-high lesion in the left middle cerebellar peduncle. Irregular ring enhancement was present.	Not performed.	Elevation of anti-nuclear, –cardiolipin, and –double-stranded DNA antibodies. Negative for anti-Hu, –Ri, –Yo, and –Ma2 antibodies.	Dexamethasone and radiation therapy to the seminoma.	Good response. 4 years later, he became asymptomatic.	Plotkin SR
4	60	M	Memory loss and homonymous right upper quadrantanopia.	Large confluent lesion affecting both occipitoparietal lobes, crossing the splenium of the corpus callosum.	No evidence of neoplasia. Demyelination, CD68-positive macrophage infiltration containing myelin debris, and scattered CD45^+^ and CD3^+^ lymphocytes.	Negative for anti Yo, –Hu, and –Ri antibodies.	Cisplatin and etoposide for seminoma. Initial steroid pulse was ineffective. Repeated steroid pulse and five plasma exchanges were added.	Partial response. Left hemianopia and memory impairment remained.	Broadfoot JR
5	62	M	Headache, right-sided weakness, and receptive aphasia.	T1-low and T2-high lesion in the left frontoparietal area with a small mass effect and gadolinium enhancement. MRS showed an NAA/choline ratio of 0.42 with a lactate peak.	No evidence of neoplasia. Demyelination and infiltration of CD68-positive foamy macrophages.	Negative for anti-Ma2, –AQP4, and –MOG antibodies.	Steroid pulse, oral steroids, and radiotherapy to the seminoma.	Poor response. Severe right hemiparesis remained.	Thebault S
6	47	M	Motor aphasia and right facial and brachial paresis.	T1-low and T2/FLAIR-high lesion that expanded through the internal capsule to the left cerebral peduncle, imcomplete ring enhancement, and visualized central veins. Mass effect was small.	No evidence of neoplasia. CD68-positive macrophage infiltration and perivascular lymphocytic infiltration.	Negative for anti–Hu, –Yo, –Ri, –CV2, –Ma1, –Ma2, –Ta, –amphiphysine, –Zic, –SOX, –GAD65, –Tr, –ANNA3, –PCA2, and –cerebellum antibodies.	Sterod pulse, oral corticosteroid, and radical orchiectomy.	Partial response. Aphasia and paresis recovered. Behavioral problems remained.	Van Haver AS
Present case	32	M	Right hemiplegia and right hemianopia.	T1-low and T2/FLAIR-high lesion in the left thalamus. Enhancement was absent. Choline, NAA, and lactate peaks on MRS.	No evidence of neoplasia. Demyelination, CD68-positive macrophage infiltration with myelin debris, and axonal damage.	Positive for anti-amphiphysin antibodies. Negative for anti-nuclear, –AQP4, and –MOG antibodies.	Radical orchiectomy, bleomycin, etoposide, and cisplatin for seminoma. Steroid pulse and gamma globulin therapy for demyelination.	Partial response. Hemiparesis and hemianopsia remained.	Shiraishi W

*ANNA, anti-neuronal nuclear antibody; AQP4, aquaporin-4; CD, cluster of differentiation; FLAIR, fluid-attenuated inversion recovery; GAD, glutamate decarboxylase; M, male; MOG, myelin oligodendrocyte glycoprotein; MRS, magnetic resonance spectroscopy; NAA, N-acetyl aspartate; PCA, Purkinje cell antibody*.

As for the prognosis of TD, two previous cases showed favorable outcomes, while four showed poor outcomes. The treatment options are generally corticosteroid therapy and treatment of seminoma. Some cases, like our patient, did not respond to the initial steroid treatment and the lesions grew in size. Plasma exchange was used in one case. We treated our case with immunotherapy along with chemotherapy. Simultaneous plasma exchange with chemotherapy is considered to reduce the effectiveness of chemotherapy (11); therefore, to treat paraneoplastic demyelination without diminishing the therapeutic effect of chemotherapy, we used a combination of corticosteroids and gamma globulin therapy. However, there is no standardized treatment option for TD. Shah et al. reported a retrospective review of the treatment of TD, showing that the patients received steroid therapy, immunomodulatory drugs, biologics, intravenous immunoglobulin, anticonvulsants, and therapeutic plasma apheresis (13). They stated that there were no correlations between treatment and disease outcome. Vakrakou et al. suggested that when corticosteroids and plasma exchange are ineffective, cyclophosphamide can be an additional treatment option for TD (14).

Our patient showed neurological symptoms 2 days after the second COVID-19 vaccination. Generally, CNS demyelination after vaccination is rare (15). Although not common, there are some reports of CNS demyelinating diseases associated with COVID-19 vaccination (16). Ismail et al. reported that all types of COVID-19 vaccines could cause CNS demyelination, and the neurological symptoms commonly appear within the first 1–2 weeks after vaccination (16). In their study, CNS demyelination after receiving the Moderna vaccine was considered to be a relapse of MS and transverse myelitis, and there are no reports of new-onset TD, which was shown in our case, after COVID-19 vaccination. In addition, the pathological features of our case did not display the hallmarks of acute disseminated encephalomyelitis (ADEM), including perivenous demyelination, fever, headache, meningitis, and consciousness disturbance (17). The lack of these symptoms in our case indicated a primary demyelinating disease rather than ADEM. Therefore, paraneoplastic TD is a more appropriate diagnosis for our case than COVID-19 vaccine-associated ADEM. However, there remains the possibility that some kind of immune response to the vaccine triggered paraneoplastic demyelination. Ismail et al. reported that among 32 cases of demyelinating disease associated with the COVID-19 vaccine, two had cancer (16). One hypothesis of the immunoreaction according to COVD-19 vaccination is molecular mimicry. The similarity between the viral proteins used for the vaccine and self-antigens (e.g., myelin) triggers an unexpected immune response (18). A previous report showed that COVID-19 infection could cause demyelination *via* decreasing T cells, B cells, or NK cells. Another demyelination mechanism is provoked auto-immune reactions, resulting from excessive self-response and antigen-driven immune responses (19). It is not clear if the COVID-19 vaccination produces a similar response. Still, the details have not been clarified. Tumefactive demyelination has been reported to be associated with an immune response (1). Therefore, autoantibodies like anti-amphiphysin antibody, and vaccination may have been involved in the pathophysiology. At present, COVID-19 is still prevalent worldwide, and further accumulation of similar cases is necessary.

To our knowledge, this is the sixth case report of pathologically confirmed paraneoplastic TD with seminoma. Our case exhibited unique and complicated pathomechanisms, including COVID-19 vaccination and anti-amphiphysin antibodies. In addition to brain biopsy pathology, peculiar MRI findings, such as a small mass effect and absence of gadolinium enhancement, and MR spectroscopy findings led us to the correct diagnosis. Finally, we administered steroid therapy and intravenous immunoglobulin, which ameliorated the disease. We hope that this case report provides helpful information for diagnosing and treating similar cases in the future.

## Data Availability Statement

The raw data supporting the conclusions of this article will be made available by the authors, without undue reservation.

## Ethics Statement

Written informed consent was obtained from the individual(s) for the publication of any potentially identifiable images or data included in this article.

## Author Contributions

WS and TU participated in patient management, clinical data analysis, and writing of the article. YN participated in patient management and revision of the article. YY, MS, and TH participated in clinical data analysis and revision of the article. All authors contributed to the article and approved the submitted version.

## Conflict of Interest

The authors declare that the research was conducted in the absence of any commercial or financial relationships that could be construed as a potential conflict of interest.

## Publisher's Note

All claims expressed in this article are solely those of the authors and do not necessarily represent those of their affiliated organizations, or those of the publisher, the editors and the reviewers. Any product that may be evaluated in this article, or claim that may be made by its manufacturer, is not guaranteed or endorsed by the publisher.
